# Assessing Complex Working Memory in Turkish-Speaking Children: The Listening Span Task Adaptation Into Turkish

**DOI:** 10.3389/fpsyg.2020.01688

**Published:** 2020-07-08

**Authors:** Gülten Ünal, Duygu Özge, Theodoros Marinis

**Affiliations:** ^1^Department of Psychology, Ankara Yıldırım Beyazıt University, Ankara, Turkey; ^2^Department of Foreign Language Education, Middle East Technical University, Ankara, Turkey; ^3^Department of Linguistics, University of Konstanz, Konstanz, Germany; ^4^School of Psychology and Clinical Language Sciences, University of Reading, Reading, United Kingdom

**Keywords:** listening span task, children, Turkish, adaptation, validity

## Abstract

The aim of this study was to adapt the Listening Span Task (Gaulin and Campbell, 1994; Pickering and Gathercole, 2001) into Turkish (LST-T), to administer it to children in order to measure how children of different ages perform on the task and to measure its psychometric properties by providing correlations with other cognitive measures: the Word Span Test that measures phonological WM capacity, the Wisconsin Card Sorting Test that measures both categorization ability and executive WM functions, and the Categorical Free Recall Test that examines both the development of the release from proactive interference pattern and the categorization ability during childhood. The results indicate that the LST-T scores increased in a significant stepwise manner without any gender difference between boys and girls. Measures of convergent validity showed significant correlations with a working memory test: the Word Span Test, and. The results demonstrate that the LST-T is an adequate tool to be used by developmentalists for a variety of purposes, ranging from developmental research to educational and clinical practice to investigate cognitive development of Turkish-speaking children.

## Introduction

From infancy to adulthood, children develop dramatically along physical, social, and cognitive dimensions. Their cognitive development critically hinges on “parameters of information processing, including working memory, the control of attention, inhibition of proponent schemes, and self-regulation (.).” (Cowan, 2016, p. 239). Working memory is commonly defined as “the small amount of information that is kept in an easily retrievable state concurrently” (Cowan, 2010, p. 447). The distinction between “storage – processing” corresponds to two types of memory span – “simple span” and “complex span,” respectively (Gathercole, 1999; Gathercole and Alloway, 2004; Cowan and Alloway, 2009; Cowan, 2010). Simple span tasks measure reproduction of verbal or non-verbal stimuli, where tasks tapping only storage are called short-term memory (STM) tasks and tasks tapping storage and processing are called working-memory (WM) tasks, in the narrow sense. Verbal STM tasks comprise the digit span, word span, and non-word repetition task, whereas non-verbal (visuospatial) STM tasks comprise pattern recall and Corsi blocks (Gathercole, 1999). Complex WM tasks comprise the backward digit span, listening span, counting span, operational span (only used with older children and adults), and (versions of) the Stroop task (Gathercole, 1999; Cowan, 2016).

Complex WM tasks crucially require the ability to control attention, i.e., executive functions, as observed in behavioral and neuroimaging research (Cowan and Alloway, 2009). In particular, the frontal lobes as well as parietal and the striatal regions are implicated in executive functions (Alvarez and Emory, 2006; Bunge and Wright, 2007). The prefrontal cortex (PFC) develops notoriously late into early adulthood due to slow myelination of nerve cells (Chugani, 1994).

Complex (but not simple) span tasks have been found to correlate highly with fluid intelligence and intellectual aptitude tasks (Cowan and Alloway, 2009; Cowan, 2010, 2016), and therefore, have become the “gold standard for the measurement of working memory” (Cowan and Alloway, 2009, p. 322). The reason behind this correlation lies in the fact that storage of the items with the help of mnemonic strategies is hindered by the intermitting processing episodes. Complex span scores, therefore, reflect a “core capacity”—devoid of any additional resource (cf. also Cowan et al., 2005). Note, however, that in children who are too young to engage in such mnemonic strategies, even a simple digit span test may correlate with aptitude (Cowan and Alloway, 2009; Cowan, 2016). In that case, i.e., when mnemonic strategies are prevented, performance in complex span tasks may depend on the “scope of attention,” which may be tantamount with “working memory capacity in chunks” (Cowan and Alloway, 2009, p. 323).

The study of complex working memory goes back to Daneman and Carpenter (1980) who devised the sentence span procedure for adults. In this task, participants read *n* sentences aloud, e.g., “When at last his eyes opened, there was no gleam of triumph, no shade of anger.” “The taxi turned up Michigan Avenue where they had a clear view of the lake” and they have to repeat the last word of each sentence (“anger, lake”). Crucially, the *n* is increased, until the participant fails in two of the trials on the respective level. The level they reach is their “reading span level.” The Daneman and Carpenter (1980) study included variants of the sentence span that involved silent reading and listening, all of which correlated with reading comprehension. Following the sentence span task, other complex measures were developed, namely the counting span (where arrays of dots need to be counted, the numbers to be stored and then, after *n* counting operations, to be recalled) and the operation span (where the result of a simple addition of two numbers needs to be stored and then, after *n* addition operations, to be recalled).

Of these three complex span tasks, the reading span task was adapted for children in the form of the listening span task by Gaulin and Campbell (1994) and by Pickering and Gathercole (2001). The procedure is slightly different from the reading span task; children listen to sentences that are considerably shorter than the sentences for adults, e.g., “Oranges are black,” they have to judge the truth-value of the sentence, and repeat the last word (for details, see “The Present Study” section).

The listening span task is highly relevant for developmentalists for the following reasons: First, it is a viable adaptation of a validated test for adults measuring working memory capacity and control, correlating highly with intellectual aptitude. Second, it is a task that can be used for a wide age range. This aspect resonates with Cowan’s suggestion to use the same methods for younger and older children in order to show the developmental increase in WM capacity – ideally already at 3 years (however, the lower bound of the listening span task seems to be around 4–5 years). Third, complex WM is not only relevant for the sake of memory itself, but is involved in a great variety of higher cognitive functions, among them reasoning, planning, goal-directed behavior, and theory of mind (ToM). Fourth, it is a capacity that shows inter-individual variation (Cowan and Alloway, 2009), allowing complex span scores to be meaningfully related to scores obtained in these other domains during development. Therefore, it is important that this instrument be available in languages other than English.

## The Present Study

The aim of the present study was to adapt the Listening Span Task (Gaulin and Campbell, 1994; Pickering and Gathercole, 2001) into Turkish (LST-T) and to administer it to monolingual Turkish children of various ages in order to measure (1) the performance of children of different ages in the task and (2) its psychometric properties, i.e., its convergent validity. To measure its convergent validity the LST-T was administered together with a working memory test: the Word Span Test that measures phonological WM capacity (Gathercole, 1999). Working memory has been shown to be positively associated with a large number of cognitive processes and abilities but not with short-term memory (Florit et al., 2009). A regression analysis was carried out to examine the predictive power of the Word Span Test, the Wisconsin Card Sorting Test, and the Categorical Free Recall Test on LST-T (total words).

## Methods

### Participants

One hundred one monolingual Turkish children (48 female) from two primary schools in Central Anatolia in Turkey participated in this study (1st Grade: *M* = 6.7, *SE* = 0.13; 2nd Grade: *M* = 7.6, *SE* = 0.09; 3rd Grade: *M* = 8.7, *SE* = 0.18; 4th Grade: *M* = 9.5, *SE* = 0.09; 5th Grade: *M* = 10.5, *SE* = 0.14).

The children attended public primary schools from the first to the fifth grade. All children had normal or corrected to normal vision and hearing, and none was reported to have any psychological or behavioral disorder. Ethical committee approval for the current study was provided by the Ethics Committee of the Middle East Technical University and parental consent was taken for each child.

### Materials, Procedure, and Scoring

We adapted Pickering and Gathercole’s (2001) Listening Span Task into Turkish. In this task, participants listen to sets of grammatical utterances, decide on the truth condition of each statement while repeating the last word of each utterance, and they recall these words at the end of each set. The number of words recalled constitutes the listening span of each participant. The test consists of 6 arrays of sets differing in size (range: 2–6), and within each array there are 6 sets.

The task includes 212 sentences. Half of these sentences were semantically plausible and the other half semantically implausible. The sentences were controlled for the number of morphemes and words. They ranged between 4 and 11 morphemes and between 2 and 3 words. Each critical word (last word in each sentence) was controlled for the morpheme length (range: 2–4). The category of the critical word was also controlled: we included an equal number of verbal, nominal, and adjectival predicates, as in (1) – see Supplementary Material for the full list of sentences.


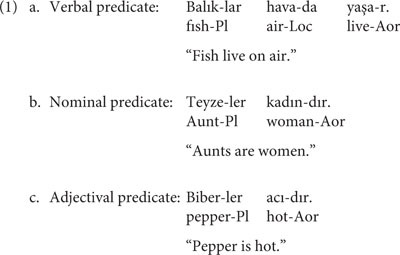


Children were tested individually in a quiet room in their school. The sentences were read by a female researcher (the first author) with a natural intonation. Each sentence was only uttered once. Children were praised at the end of each set regardless of their performance. The set size of the sentences increased in line with the children’s performance. If children made two or more mistakes in a set, the test was terminated.

The total number of words correctly recalled (Total words) and the longest set size in which the child recalled all the words in at least 4 out of 6 trials (Total level) are the two main scores of this test. In addition, the total number of questions answered correctly (Listening comprehension) was an additional score.

Apart from the adapted LST-T, children completed one working memory tests, i.e., the Word Span Test (WST; Pickering and Gathercole, 2001), and the Wisconsin Card Sorting Test (WCST; Cianchetti et al., 2007), and a short-term memory test, the Categorical Free Recall Test (CFRT; Halford et al., 1994) so that we can measure convergent validity (see Supplementary Material for the descriptions and the descriptive statistics of these additional tasks). Each child was tested in a quiet room inside the school she attended in one session lasting ~20 min each.

## Challenges in Adapting LST-T Into Turkish

The adaptation of the task from English, an Subject-Verb-Object language with poor morphology and with strict word order, to Turkish, an Subject-Object-Verb language with rich morphology and word order variation, created several challenges. The first and most important issue stemmed from the word-order differences between English and Turkish. In the English version, the final word to be recalled naturally varied from sentence to sentence. However, since Turkish generally locates the verb in the sentence-final position, we needed to vary the type of the predicate to avoid having a verbal predicate all the time. In order to balance the type of the final word, we used an equal number of verbal, nominal, and adjectival predicates, as in the examples in (1) above. The second major challenge was related to the rich concatenative morphology in Turkish. In the English version, the final word of each sentence was not morphologically inflected (e.g., “Peppers are hot”), the Turkish version included predicates marked in the number, person and the aorist morpheme expressing objective predication. To control for the morphological complexity, we balanced the number of morphemes in each last word. We believe that this did not create any extra difficulty for our participants, since the sentences were syntactically simple and were the type of sentences children hear in their everyday life. Finally, in order to ensure cultural appropriateness, we eliminated one sentence about pigs (e.g., “Pigs have curly tails”) because our participants may have never seen a pig, making the truth judgment difficult. We also eliminated one more sentence (“Fathers are men”) to eliminate the gender bias.

## Results

Some children from the lower grades had difficulties to understand what “the last word of a sentence” meant and needed training on this concept. After training, they were able to understand this concept and were able to complete the task. Table 1 shows the descriptive statistics of the LST-T task.

**TABLE 1:** Descriptive statistics for the LST-T (level and total words recalled).

**Grades**	***N***	**Mean level (out of 6)**	**SD level**	**Mean total words**	**SD total words**
1st Grade	20	0.70	0.66	2.05	1.88
2nd Grade	24	2.02	0.31	5.54	2.15
3rd Grade	16	2.00	0.52	6.19	3.21
4th Grade	22	2.73	0.37	12.05	3.71
5th Grade	19	2.74	0.30	12.05	2.35

The children’s scores increased in a stepwise manner but not linearly. A Kruskal-Wallis Test on the total words recalled showed that the children’s scores were affected by grade [Kruskal-Wallis Test, χ*^2^*(4) = 69.97, *p* < 0.001]. There was a significant increase in the children’s scores between the 1st (*M* = 2.05, *SD* = 1.88) and 2nd grade (*M* = 5.54, *SD* = 2.15) and between the 3rd (*M* = 6.19, *SD* = 3.21) and 4th grade (*M* = 12.05, *SD* = 3.71). There was no significant effect of gender (*Z* = -0.28, *p* < 0.05).

A similar analysis on the level showed a similar pattern for the children’s scores regarding the span level. A Kruskal-Wallis Test on the level of the LST-T showed that the children’s scores were affected by grade [Kruskal-Wallis Test, χ*^2^*(4) = 74.75, *p* < 0.001]. There was a significant increase in the children’s scores between the 1st (*M* = 0.70, *SD* = 0.66) and 2nd grade (*M* = 2.02, *SD* = 0.31) and between the 3rd (*M* = 2.00, *SD* = 0.55) and 4th grade (*M* = 2.76, *SD* = 0.37). There was no significant effect of gender for the level of LST-T (*Z* = -0.14,*p* < 0.05).

To measure the LST-T’s convergent validity we conducted Spearman’s Correlations between the children’s scores on the LST-T and their scores on the Word Span Test, and the absolute order of items of the Categorical Free Recall Test. Additionally, Spearman’s partial correlations analyses controlling for age were conducted to address whether the correlations will still be present if we control for age. The correlations and partial correlations are presented in Table 2.

**TABLE 2:** Spearman’s correlations among the LST-T and the other cognitive measures.

		**1**	**2**	**3**	**4**	**5**	**6**
1.	CT-absolute order of items		0.291*	0.208*	0.262*	0.239*	0.319**
2.	CT-total recalled items	259*		0.414**	0.310*	0.253*	0.194
3.	WST	0.084	0.388**		0.465**	0.493**	0.504*
4.	WCST	0.137	0.267*	0.256*		0.572**	0.548**
5.	LST-T-level	0.063	0.196*	0.217*	0.296*		0.905**
6.	LST-T-total recalled words	0.177	0.107	0.216*	0.230*	0.795**	

N = 101; ** stands for p < 0.01; * stands for p < 0.05; CT, Categorical Free Recall Test; WST, Word Span Test; WCST, Wisconsin Card Sorting Test; LST-T, Listening Span Test-Turkish. Top triangle indicates bivariate correlations/bottom triangle indicates partial correlations.

There were significant moderate correlations between the LST-T scores (both level and total words) and the scores on the Word Span Test (Word Span Test: 0.493; 0.504). Moreover, there were weak correlations between the LST-T scores (both level and total words) and the absolute order of items on the Categorical Free Recall test (0.239; 0.319), as well as between the LST-T scores (level) and the total number of recalled items on the Categorical Free Recall test (0.253). Importantly, there was no significant correlation between the total number of recalled words on the LST-T and the total number of recalled items on the Categorical Free Recall test (0.194). The majority of the results of the partial correlations are significant after controlling for age.

The Word Span Test, the Wisconsin Card Sorting Test, and the Categorical Free Recall Test (total number of recalled items in absolute order) scores predicted the LST-T (total words recalled) [*R*^2^ = 0.40, *F*(1, 97) = 21.79, *p* < 0.001]. Multiple regression results indicated that the Wisconsin Card Sorting Test [*ß* = 0.366, *t*(97) = 4.05, *p* < 0.001] was a significant predictor (among other predictors: Word Span Test (*ß* = 0.300, *t*(97) = 3.37, *p* = 0.001) and the Categorical Free Recall Test [*ß* = 0.160, *t*(97) = 1.96, *p* = 0.053)] for the total number of recalled words in LST-T.

## Discussion and Conclusion

Although the LST is a highly relevant task for researchers conducting studies on language development, there has been a lack of an LST for Turkish speaking children. This motivated us to develop a Turkish LST and validate it with Turkish-speaking children in primary schools in Turkey. The LST-T had to accommodate the typological properties of Turkish: word-order, distribution of grammatical classes, and rich concatenative morphology.

The LST-T was linguistically and culturally adequate for Turkish primary school children. The children were able to complete the task when training was provided about the concept “the last word of the sentence.”

The performance rates of 101 monolingual Turkish children between the ages of 5; 6 and 12; 0 showed that complex working memory significantly increases with age and school grade. These results are consistent with similar studies in which the LST score increases significantly during childhood (for instance, Naito, 2003; Gathercole et al., 2004; for the relation between memory and listening comprehension, see Florit et al., 2009).

Correlational analyses between the results of the LST-T and the Word Span Test and the Categorical Free Recall Test yielded significant convergent validity results. There were significant correlations between the LST-T, the Word Span Test, and the absolute order of items on the Categorical Free Recall test, but the correlations between the LST-T and the absolute order of items of the Categorical Free Recall Test were not significant. This demonstrates that the LST-T reflects the pattern of performance in other working memory and executive function measures (the Word Span Test, the Wisconsin Card Sorting Test, and the absolute order of items on the Categorical Free Recall test) while not reflecting the short term memory performance (the absolute order of items of the Categorical Free Recall Test). This strengthens its the validity and suggests that the Turkish version of the LST, just like the English version, successfully taps into aspects of working memory functioning. The fact that the LST-T was not correlated with the short term memory measure is consistent with Baddeley and Hitch’s (1974) model of working memory, positing the existence of a central executive that is working independently of the two subsystems of the phonological loop and the visuo-spatial sketchpad. That is, while the LST-T taps into the central executive as well as the phonological loop, the absolute order of items of the Category Recall Task might be tapping only into phonological memory.

The results of this study are important for developmentalists because working memory capacity is predictive of many other developmental functions, such as problem solving (Swanson and Beebe-Frankenberger, 2004), reading comprehension (Cain et al., 2004), mental arithmetic (Adams and Hitch, 1997), language development (Gathercole, 2006), sentence processing (Montgomery, 2000), and academic performance (Gathercole et al., 2003; Pickering and Gathercole, 2004). Therefore, we believe that the LST-T is an adequate tool to be used for a variety of purposes, ranging from developmental research to educational and clinical practice to investigate the cognitive development of Turkish-speaking children.

The research presented in this paper consists of a small-scale study. Future research should aim to replicate the findings with a larger sample, extend the data to younger and older children, and develop norms across a range of age groups in line with Pickering and Gathercole (2004). Within such a large-scale study, it would be possible to create a second version of the task in order to be able to measure test-retest reliability and to be able to use the task for pre-post evaluations. This was not done in the present study, and therefore, it was not possible to obtain reliability estimates (e.g., test-retest reliability), which is a limitation of the study. Such a large-scale study could also address one of the limitations of the procedure used in LSTs, i.e., the fact that the number of trials administered may vary between individuals because of the discontinuation rule. As a result, different children can finish the task with different levels of fatigue which may induce different levels of proactive interference. This is important, especially if the task is used in combination with other tasks. This issue could not be addressed within the remit of the present study and remains open for future research.

## Data Availability Statement

The datasets generated for this study are available on request to the corresponding author.

## Ethics Statement

The studies involving human participants were reviewed and approved by Middle East Technical University Applied Ethical Research Center. Written informed consent to participate in this study was provided by the participants’ legal guardian/next of kin.

## Author Contributions

GÜ: background of the study, statistical analysis, data collection and interpretation, writing of the article. DÖ and TM: concept of the study, adaptation of the LST, data interpretation, writing of the article, and proofreading. All authors contributed to the article and approved the submitted version.

## Conflict of Interest

The authors declare that the research was conducted in the absence of any commercial or financial relationships that could be construed as a potential conflict of interest.
