# Supplementary material for: Assessing Complex Working Memory in Turkish-Speaking Children: The Listening Span Task Adaptation Into Turkish
Source: Front Psychol. 2020 Jul 8;11:1688. doi: 10.3389/fpsyg.2020.01688 (PMC7360717; doi:10.3389/fpsyg.2020.01688)
Supplement: Supplementary file 1 [file Presentation_1.pdf]

Supplementary Material - I

Turkish Listening Span Test (LST-T)

Used in the trials

1. Çocuklar okula gider.
2. Balıklar havada yaşar.
3. Ağaçlar dans eder.

Sets with 2 Sentences

1

1. Biber acıdır.
2. Kediler okulda çalışır.

2

1. Filler çok küçüktür.
2. Ayakkabı ayağa giyilir.

3

1. İnsanlar saçlıdır.
2. Çicekler fare kovalar.

4

1. Ayılar araba sürer.
2. Havuçlar turuncudur.

5

1. Gece karanlıktır.
2. Portakallar suda yaşar.

6

1. Ateş sıcaktır.
2. Balıklar konuşur.

Sets with 3 Sentences

1

1. Otobüslerle tatile gideriz.
2. Toplar karedir.
3. Öğretmenler ağaçta yetişir.

2

1. Muzlar bisiklete biner.
2. Elimiz beş parmaklıdır.
3. Soğan acıdır.

3

1. Otobüsler oyuncakla oynar.
2. Kuşlar kanatlıdır.
3. Elmalar ağaçta yetişir.

4

4. Piyanolar müzik çalar.
5. Kardeşlerimiz kuyrukludur.
6. Burnumuzla görürüz.

5

4. Ayağımız çenelidir.
5. Güneş sıcaktır.
6. Taşlar serttir.

6

4. Kaşıklarla yazı yazarız.
5. Limon sarıdır.
6. Köpekler kedileri kovalar.

Sets with 4 Sentences

1

1. Zürafalar uzun boyludur.
2. Çiçekler pasta sever.
3. Portakallar kulaklıdır.
4. Öğretmenler okulda çalışır.

2

1. Otobüsler konuşur.
2. Bankalardan para çekeriz.
3. Kışlar sıcaktır.
4. Pastalar tatlıdır.

3

1. Gökyüzü kırmızıdır.
2. Bebekler ağlar.
3. Köpekler konuşur.
4. Muzlar tatlıdır.

4

1. Armutlar mavidir.
2. Şapkalar başa giyilir.
3. Tavşanlar saati gösterir.
4. Filler büyüktür.

5

1. İnsanlar iki ayaklıdır.
2. Portakallar siyahtır.
3. Kediler futbol oynar.
4. Kitapları okuruz.

6

1. Tavşanlar ağaçta yetişir.
2. Biberler yeşildir.
3. Portakallar markette satılır.
4. İnsanlar üç gözlüdür.

Sets with 5 Sentences

1

1. Babalar kanatlıdır.
2. Dondurma soğuktur.
3. Portakallar gitar çalar.
4. Arabalar benzinle çalışır.
5. Fareler çok büyüktür.

2

1. Havuçlar mavidir.
2. Kulaklarımızla görürüz.
3. Portakallar turuncudur.
4. Tavuklar yumurta yapar.
5. Bıçak keskindir.

3

1. Elmalar pembedir.
2. Karıncalar yavaştır.
3. Dondurma sıcaktır.
4. Kediler fare kovalar.
5. Bebekler tüylüdür.

4

1. Kuşlar kocamandır.
2. Motorsikletler havlar.
3. Bıçaklar yumuşaktır.
4. Bulutlar beyazdır.
5. Tavuklar yazı yazar.

5

1. Gemiler uçar.
2. Kareler yuvarlaktır.
3. Çorabı ayağımıza giyeriz.
4. Bisikletler süt içer.
5. İnsanlar iki kulaklıdır.

6

1. Uçaklar kanatlıdır.
2. Elmalar şarkı söyler.
3. Dağlar çok küçüktür.
4. Sandalyeler ayaklıdır.
5. Makaslar kağıt keser.

Sets with 6 Sentences

1

1. Muzlar dişlidir.
2. Köpekler gitar çalar.
3. Bacağımız parmaklıdır.
4. Mektupları pulla göndeririz.
5. Muzlar sarıdır.
6. Kurbağalar zıplar.

2

1. Oyuncak ayılar yumuşaktır.
2. Ördekler suda yaşar.
3. Çocuklar üç kolludur.
4. Evimiz şarkı söyler.
5. Ördekler beş ayaklıdır.
6. Kar soğuktur.

3

1. Saatler zamanı gösterir.
2. Ayran tatlıdır.
3. Kurbağalar uzun kulaklıdır.
4. Ağaçlar müzik çalar.
5. Toplar yuvarlaktır.
6. Balıklar suda yaşar.

4

1. Arılar sokar.
2. Koyunlar kuyrukludur.
3. İnekler uçar.
4. Köpek balığı kocamandır.
5. Bulutlar siyahtır.
6. Pamuk ağırdır.

5

1. Ağaçlar tüylüdür.
2. Marketler yiyecek satar.
3. Domates kırmızıdır.
4. Kediler çok büyüktür.
5. Tavşanlar uzun kulaklıdır.
6. Tavuklar okula gider.

6

1. Kirazlar mavidir.
2. Ağaçlar yapraklıdır.
3. Demir hafiftir.
4. Yılanlar zıplar.
5. Kekler tatlıdır.
6. Tekerlekler karedir.

Supplementary Material - II

Descriptive Statistics for the Additional Tasks

Table 1. Descriptive statistics for the Categorical Free Recall Test

	N	*Mean*[1]	*S.D.*[1]	*Mean*[2]	*S.D.*[2]
1st Grade	20	4.85	2.08	.70	.98
2nd Grade	24	5.46	.93	.79	1.10
3rd Grade	16	5.88	1.26	.94	.99
4th Grade	22	6.05	.79	1.50	1.26
5th Grade	19	5.63	1.46	1.47	1.17

*Note.*1 means “total words recalled”, and 2 means “total number of absolute order of recalled words”.

Table 2. Descriptive statistics for the Word Span Test for all grades

	N	*Mean*	*S.D.*
1st Grade	20	3.33	1.03
2nd Grade	24	3.71	.69
3rd Grade	16	4.13	.62
4th Grade	22	4.136	.47
5th Grade	19	4.37	.60

Table 3. Descriptive statistics for the Wisconsin Card Sorting Test for all grades

	N	*Mean*	*S.D.*
1st Grade	20	18.25	9.72
2nd Grade	24	24.79	5.58
3rd Grade	16	27.19	6.04
4th Grade	22	30.23	7.62
5th Grade	19	31.95	3.44

Supplementary Material - III

Descriptions of the Additional Tasks

Categorical Free Recall Test

Previously learned information may have proactive interference with newly learned information. But if this information is collected under different categories, release from proactive interference may occur (Darling & Valentine, 2005). Eliminating proactive interference could be measured with the help of a categorical short-term memory test. The aim of the Categorical Free Recall Test is to both measure release from proactive interference and to measure short-term memory span. In this test, the participant listens to stimuli from different categories and is required to recall these stimuli in the given order. The release from proactive interference occurs between the last item of each category and the first item of the next category. In this task, there were 3 categories (fruits, animals, and clothes) and 12 words (4 words from each categories such as grape, chicken, and shirt). Eighteen different orders of items and categories were used. Each child only listened to one of these orders once. After having listened to the items, the child were required to tell what s/he recalled from the list in serial order. The main dependent variables of the study are (i) total number of recalled items (without any order), and (ii) total number of recalled items in absolute order.

Word Span Test

The "Word Recall Test" of the Working Memory Test Battery for Children (Pickering & Gathercole, 2001, as cited in Alloway, Gathercole, Willis, & Adams, 2004) was translated and adapted into Turkish and administered to children as "Word Span Test". In this task, the child is asked to recall a set of words in the presented order. The set size increases by one until the child makes a mistake on one of the four trials in each level. The word span is calculated by the correctly recalled number of trials.

Wisconsin Card Sorting Test

The Wisconsin Card Sorting Test (WCST) is a complex memory measure (Cianchetti, Corona, Foscoliano, Contu, & Sannio-Fancello, 2007). It is "a complex task involving learning, elaboration of strategies for hypothesis testing and problem-solving" (Stratta, Daneluzzo, Prosperini, Bustini, Mattei, & Rossi, 1997, p.18). This test was used to measure both the ability to categorize and the executive working memory functions. It includes two sets of 24 cards including different combinations of colors (yellow, red, green, blue), numbers (one, two, three, four), and forms (triangle, star, circle, cross). In the test, the child is given four stimulus cards

(a single red triangle, two green stars, three yellow crosses, and four blue circles) and is asked to indicate one of these cards when she is given the response cards one by one. Six consecutive response cards are belong to one of these categories (color, form or number). The child is expected to find these categories and to have the ability to make a shift among these categories. The dependent variable of this study is the total number of correct responses.

References

Alloway, T. P., Gathercole, S. E., Willis, C., & Adams, A.-M. (2004). A structural analysis of working memory and related cognitive skills in young children. *Journal of Experimental Child Psychology*, *87*(2), 85-106. doi: 10.1016/j.jecp.2003.10.002

Cianchetti, C., Corona, S., Foscoliano, M., Contu, D., & Sannio-Fancello, G. (2007). Modified Wisconsin Card Sorting Test (MCST, MWCST): Normative data in children 4–13 years old, according to classical and new types of scoring. *The Clinical Neuropsychologist*, *21*(3), 456 - 478. doi:10.1080/13854040600629766

Darling, S., & Valentine, T. (2005). The categorical structure of semantic memory for famous people: a new approach using release from proactive interference. *Cognition*, *96*(1), 35-65. doi: 10.1016/j.cognition.2004.03.007

Pickering, S. J., & Gathercole, S. E. (2001). *Working memory test battery for children*. London: Psychological Corp.

Stratta, P., Daneluzzo, E., Prosperini, P., Bustini, M., Mattei, P., & Rossi, A. (1997). Is wisconsin card sorting test performance related to ‘working memory’ capacity? *Schizophrenia Research*, *27*(1), 11-19. doi: 10.1016/S0920-9964(97)00090-X
